# Molecular Docking and Molecular Dynamics Studies Reveal the Anticancer Potential of Medicinal-Plant-Derived Lignans as MDM2-P53 Interaction Inhibitors

**DOI:** 10.3390/molecules28186665

**Published:** 2023-09-16

**Authors:** Tagyedeen H. Shoaib, Nihal Abdelmoniem, Rua M. Mukhtar, Amal Th. Alqhtani, Abdullah L. Alalawi, Razan Alawaji, Mashael S. Althubyani, Shaimaa G. A. Mohamed, Gamal A. Mohamed, Sabrin R. M. Ibrahim, Hazem G. A. Hussein, Abdulrahim A. Alzain

**Affiliations:** 1 Department of Pharmaceutical Chemistry, Faculty of Pharmacy, University of Gezira, Wad Madani 21111, Sudan; shoaibth37@hotmail.com (T.H.S.); nihal.khunaijir@gmail.com (N.A.); ruamubarak1@gmail.com (R.M.M.); 2 Pharmaceutical Care Services, Madinah Cardiac Center, MOH, Al Madinah Al Munawwarah 11176, Saudi Arabia; amtalqhtani@moh.gov.sa (A.T.A.); maalthubyani@moh.gov.sa (M.S.A.); 3 Pharmaceutical Care Services, King Salman Medical City, MOH, Al Madinah Al Munawwarah 11176, Saudi Arabia; abdullahalalawi96@gmail.com; 4 Department of Pharmacology and Toxicology, College of Pharmacy, Qassim University, Qassim 51452, Saudi Arabia; razanalawaji@gmail.com; 5 Faculty of Dentistry, British University, El Sherouk City, Suez Desert Road, Cairo 11837, Egypt; shaimaag1973@gmail.com; 6 Department of Natural Products and Alternative Medicine, Faculty of Pharmacy, King Abdulaziz University, Jeddah 21589, Saudi Arabia; gahussein@kau.edu.sa; 7 Preparatory Year Program, Department of Chemistry, Batterjee Medical College, Jeddah 21442, Saudi Arabia; 8 Department of Pharmacognosy, Faculty of Pharmacy, Assiut University, Assiut 71526, Egypt; 9 Preparatory Year Program, Batterjee Medical College, Jeddah 21442, Saudi Arabia; hazemgamal2005@gmail.com

**Keywords:** cancer, MDM2-P53, PPI, Ferula sinkiangensis, *Justicia procumbens*, lignans, molecular docking, ADME, molecular dynamics, drug discovery, life on land, health and wellbeing

## Abstract

The interaction between the tumor suppressor protein p53 and its negative regulator, the MDM2 oncogenic protein, has gained significant attention in cancer drug discovery. In this study, 120 lignans reported from Ferula sinkiangensis and *Justicia procumbens* were assessed for docking simulations on the active pocket of the MDM2 crystal structure bound to Nutlin-3a. The docking analysis identified nine compounds with higher docking scores than the co-crystallized reference. Subsequent AMDET profiling revealed satisfactory pharmacokinetic and safety parameters for these natural products. Three compounds, namely, justin A, 6-hydroxy justicidin A, and 6′-hydroxy justicidin B, were selected for further investigation due to their strong binding affinities of −7.526 kcal/mol, −7.438 kcal/mol, and −7.240 kcal/mol, respectively, which surpassed the binding affinity of the reference inhibitor Nutlin-3a (−6.830 kcal/mol). To assess the stability and reliability of the binding of the candidate hits, a molecular dynamics simulation was performed over a duration of 100 ns. Remarkably, the thorough analysis demonstrated that all the hits exhibited stable molecular dynamics profiles. Based on their effective binding to MDM2, favorable pharmacokinetic properties, and molecular dynamics behavior, these compounds represent a promising starting point for further refinement. Nevertheless, it is essential to synthesize the suggested compounds and evaluate their activity through in vitro and in vivo experiments.

## 1. Introduction

Cancer is a significant global health challenge and ranks as the second leading cause of death worldwide, surpassed only by heart disease [1]. The American Cancer Society (ACS) reports that cancer is a major contributor to morbidity and mortality, with an estimated 1.9 million new cases and 609,820 cancer-related deaths expected in the United States in 2023 [2].

Protein–protein interactions (PPIs) play a critical role in various biological functions, such as cell signal transduction and DNA synthesis. These interactions have the capacity to either stimulate or prevent the occurrence, progression, and metastasis of cancer [3]. Consequently, targeting PPIs holds promise as a method for cancer treatment [4]. In particular, the interaction between MDM2 (mouse double minute 2) and p53 proteins is a major focus in the discovery and development of anticancer medications [5]. p53, known as the genome’s guardian and a tumor suppressor protein (TP53), activates in response to cellular stress and influences the transcription of numerous downstream genes involved in cell cycle regulation, apoptosis, DNA repair, and senescence [6,7,8]. Approximately 50% of all human malignancies advance due to p53 deletion or mutation [9]. Therefore, activating the pro-apoptotic protein p53 is a highly desired strategy for cancer therapy [10].

MDM2, found overexpressed in many tumor tissues, functions as the primary negative regulator of p53 [11,12,13]. Studies have revealed an autoregulatory feedback loop between p53 and MDM2, where they control each other’s activity within cells [14,15]. MDM2 binds to p53, inhibiting its transcriptional activity; shuttles the p53 protein to the cytoplasm; and subsequently targets it for degradation through the E3 ubiquitin pathway. This pathway offers an effective approach for the targeted treatment of various malignancies [16]. Consequently, the use of MDM2-p53 PPI inhibitors has emerged as a promising strategy for treating human cancers [17].

In recent years, numerous highly selective and potent small-molecule MDM2 inhibitors have been discovered, with nine of them undergoing clinical trials for cancer treatment [18].

Natural metabolites have shown tremendous promise for developing chemotherapeutic agents due to their extensive structural diversity and favorable pharmacological and molecular properties [19,20]. The majority of naturally occurring active pharmaceuticals, particularly those derived from plants, account for 75% of anticancer therapies [21,22]. Impressively, many of the registered anticancer molecules are either natural products or natural-product-based analogs [20,23], for example, Velban^®^ (Vinblastine), Taxotere^®^) (Docetaxel), Taxol^®^ (Paclitaxel), and Oncovin^®^ (Vincristine) [20,22].

**Figure 1:**
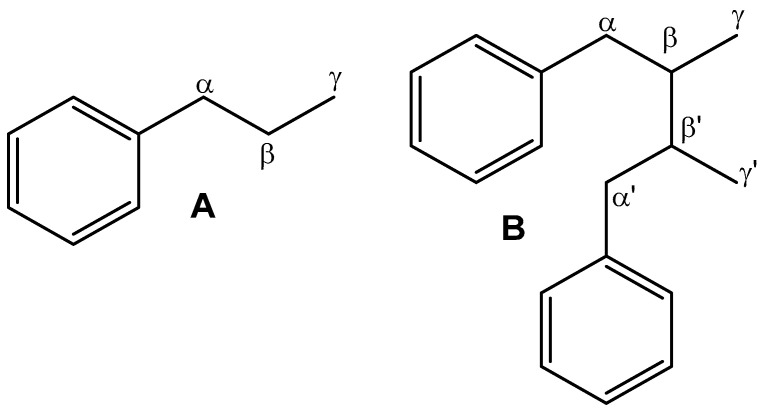
Phenyl propane moiety (**A**) and basic skeleton of lignans (**B**).

Lignans are among the phenolic components that are widely distributed in the plant kingdom and identified in various parts of over 60 plant families [24,25,26]. Structurally, lignans consist of two phenylpropane units linked by β,β-bonds (Figure 1) [27].

These metabolites possess various positive health effects, such as antiplatelet aggregation; antimicrobial, antiviral, antioxidant, anti-estrogenic, and antimutagenic activities; and marked anticancer activity against various cancerous cells [28,29,30]. Additionally, these compounds have demonstrated structural similarity to podophyllotoxin, a renowned potential antitumor metabolite. Therefore, various studies explored the biological potential and possible action mechanisms by which lignans exert their anticancer effectiveness. In this regard, the reported lignans from Ferula sinkiangensis and *Justicia procumbens* [30,31] (Supplementary Table S1) were assessed as valuable sources for the development of novel anticancer therapeutics [32]. 

It is noteworthy that *J. procumbens* and *F. sinkiangensis* are utilized as herbal remedies for treating various ailments in different countries. *J. procumbens* is employed for cancer, lumbar pain, fever, chronic glomerulonephritis, venereal and skin diseases, aphthous ulcer, sore throat, diabetes, headache, arthritis, inflammation, and gastrointestinal disorders, as well as to promote digestion, urination, and blood circulation and relieve dyspepsia [31,33,34]. In contrast, *F. sinkiangensis* is often utilized for indigestion, lumps, joint pain, baldness, bronchitis, wound infection, ovarian cysts, parasite-caused malnutrition, stomachic and abdominal swelling pain, malaria, diarrhea, abdominal mass, cold, dysentery, and measles [30,35].

Computational techniques have been employed to facilitate the discovery of potential inhibitors targeting MDM2 [36]. Among these techniques, in silico structure-based drug discovery, which includes molecular docking, molecular dynamics (MD) simulations, MM-GBSA calculations, and ADMET prediction, has been extensively utilized [37,38].

In this study, we employed in silico approaches, including molecular docking, MD simulations, MM-GBSA calculations, and ADMET prediction, to screen a library of 120 lignan compounds in search of potential MDM2 inhibitors. Our objective was to identify novel candidates with inhibitory activity against MDM2, expanding the possibilities for future therapeutic development.

## 2. Results and Discussion

**Figure 2:**
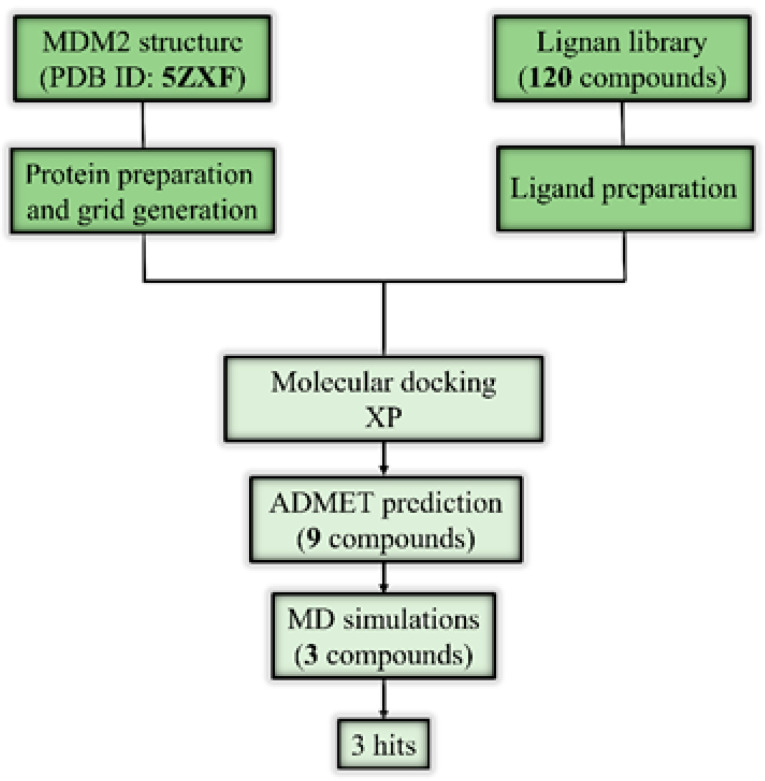
The workflow of this study shows the adopted computational approaches.

The workflow of this study is depicted in Figure 2.

### 2.1. Molecular Docking and ADMET Profiling

**Figure 3:**
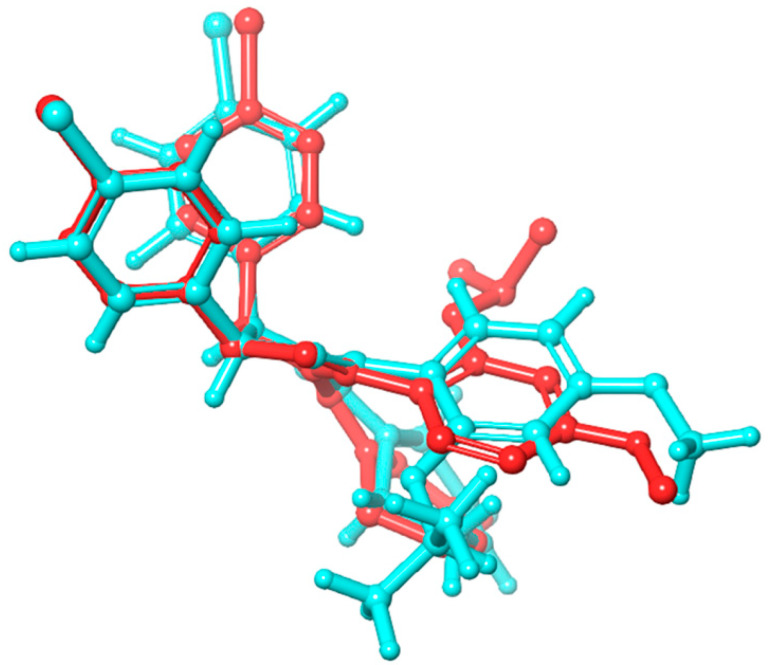
The superposition of the raw co-crystalized Nutlin-3a (red) and the XP-docked Nutlin-3a (cyan).

To date, countering MDM2-p53 pathways remains a challenging task when introducing a new anticancer agent. In this study, we focused on docking a library of 120 lignans against MDM2 (PDB ID 5ZXF). The docking procedure employed the Gide extra-precision mode, known for providing highly accurate and precise estimates of binding affinity for docked complexes [39,40,41]. Initially, the docking protocol was validated by calculating the root-mean-square deviation (RMSD) for Nutlin-3a between its co-crystallized pose before docking and the resultant docking pose. It is evident that good docking complexes correlate with an RMSD of <2.0 Å. However, docking systems with an RMSD between 2.0 Å and 3.0 Å depart from the reference’s location while maintaining the desired orientation and are deemed acceptable. At the very least, docking systems with an RMSD > 3.0 Å are incorrect in all aspects [42,43]. The reference Nutlin-3a showed an RMSD value of 1.92 Å, which is in the good range of deviation. Therefore, since the docking pose of the reference was validated, the posing pattern of the rest molecules was compared and filtered in association with this valid pose. The superposition poses of Nutlin-3a are illustrated in Figure 3. 

The docking scores obtained in the extra-precision mode ranged from −7.866 kcal/mol to −6.831 kcal/mol for nine inhibitors, surpassing the docking score of the co-crystallized reference Nutlin-3a (−6.830 kcal/mol), as shown in Table 1. Lower docking scores indicate stronger binding interactions [44,45], thus implying that these top nine inhibitors exhibited a higher affinity for binding to MDM2 than the co-crystallized inhibitor. Further analysis of the binding pattern revealed that the top hits, along with the reference inhibitor, were stabilized in the active site through various types of interactions, including hydrophobic contacts, hydrogen bonding, and π-π stacking. The active site of MDM2 contains a hydrophobic cavity formed by key amino acids, which serve as a hotspot pocket for inhibitor binding. Specifically, our analysis of the crystal structure identified nine amino acid residues (LEU-33, LEU-36, ILE-40, MET-41, PHE-65, PHE-70, VAL-72, ILE-78, and TYR-79) involved in hydrophobic interactions with the studied inhibitors. Among these amino acids, VAL-72 and LEU-33 formed hydrophobic contacts with all the top-docked complexes, while the remaining residues exhibited a variable number of interactions with the ligands, with a minimum of three interactions, as indicated in Table 1. The distances of these interactions fell within the range of 4 Å, which is considered significant for the occurrence of hydrophobic bonding.

Regarding direct hydrogen bonding, we observed interactions involving specific amino acids (LEU-33, GLN-51, HIS-52, VAL-72, and TYR-79). Additionally, water-mediated hydrogen bonds (water bridges) were observed with procumbiene, lariciresinol, and the reference Nutlin-3a, involving amino acid residues PHE-34 and GLN-38. Both types of hydrogen bonds play a significant role in ligand binding within the MDM2 pocket.

Furthermore, we observed another type of interaction, π-π stacking, which contributed to the stabilization of the top-docked complexes within the binding pocket. The amino acids HIS-75 and TYR-79 were involved in the formation of π-π stacking interactions with two hit compounds: (+)-sinkianlignan J and procumbiene.

An extensive analysis of the binding pattern revealed that the binding mode of Nutlin-3a aligns with previous findings from X-ray crystallography. The two chlorophenyl entities and the isopropoxy functional group of Nutlin-3a deeply penetrate the hydrophobic binding pocket of MDM2 [46]. Nutlins, as a class of chemical compounds, have been extensively studied as MDM2 inhibitors and have shown significant efficacy in suppressing human tumors [46,47,48]. Nutlin-3a, in particular, is one of the most recent and potent Nutlins, with an IC_50_ of 90 nM. Moreover, one drawback of Nutlin-3a is its possession of chiral centers, which complicates its synthesis and purification. In contrast, the studied compounds are of natural origin, demonstrate a better binding affinity than Nutlin-3a, and can be extracted from their sources without the challenges associated with chemical synthesis and purification.

Using the QikProp tool in Maestro, we predicted the pharmacokinetic parameters of compounds that showed better docking scores than the co-crystallized inhibitor. This step was taken to assess the druggability of these top-docked compounds and investigate their potential success in meeting Lipinski’s rule of five criteria. According to this rule, a molecule is considered druggable if it adheres to the following constraints: a molecular weight of less than 500 Da, hydrogen bond donors (donorHB ≤ 5), hydrogen bond acceptors (acceptorHB ≤ 10), and a predicted octanol/water partition coefficient (QPlogPo/w) of less than 5 [49,50].

Table 2 confirms that the co-crystallized reference inhibitor Nutlin-3a violated two parameters of the rule. Its molecular mass of 581 Da exceeds the limit set by the rule (Mwt < 500). Additionally, the predicted partition coefficient was 5.098, slightly higher than the accepted value (QPlogPo/w < 5). However, the natural product inhibitors did not violate any rule parameters. Specifically, the number of hydrogen bond donors and acceptors fell within the acceptable range, indicating their ability to form proper hydrogen bonds with the target residues. Moreover, the partition coefficient (QPlogPo/w) was within the acceptable range, suggesting the drugability of these candidate compounds. Furthermore, the predicted solubility parameter for all the compounds fell within the standard range, indicating their optimal solubility.

Two parameters related to the ability to cross cellular membranes were also examined: QPlogBB (blood–brain barrier permeability) and QPP Caco-2 (cell membrane permeability). Notably, all the compounds showed optimal cellular permeability and a limited ability to cross the blood–brain barrier. Another important parameter, QPlog HERG, which predicts the IC_50_ values for the blockage of HERG K+ channels (summarized in Table 2), indicated that none of the molecules exhibited cardiotoxicity. Additionally, the candidate molecules demonstrated a high percentage of human oral absorption, indicating excellent bioavailability.

The selection criteria for further processing the hits were based on a docking score of less than −7 kcal/mole and a percentage of human oral absorption above 95%. Applying these standards filtered out three compounds: justin A, 6′-hydroxy justicidin A, and 6′-hydroxy justicidin B. According to the LOTUS database [51], justin A, 6′-hydroxy justicidin A, and 6′-hydroxy justicidin B are natural products found in *Justicia procumbens*. Among them, 6′-hydroxy justicidin A is reported to have antitumor activity [52,53] and a patent for the prevention of coronavirus [54]. Similarly, 6′-hydroxy justicidin B is also patented for the prevention or treatment of SARS-CoV-2 infectious disease [55]. These selected natural lignans exhibited a favorable binding energy and desirable ADMET profiles, and, therefore, they were chosen for subsequent molecular dynamics simulations. 

**Figure 4:**
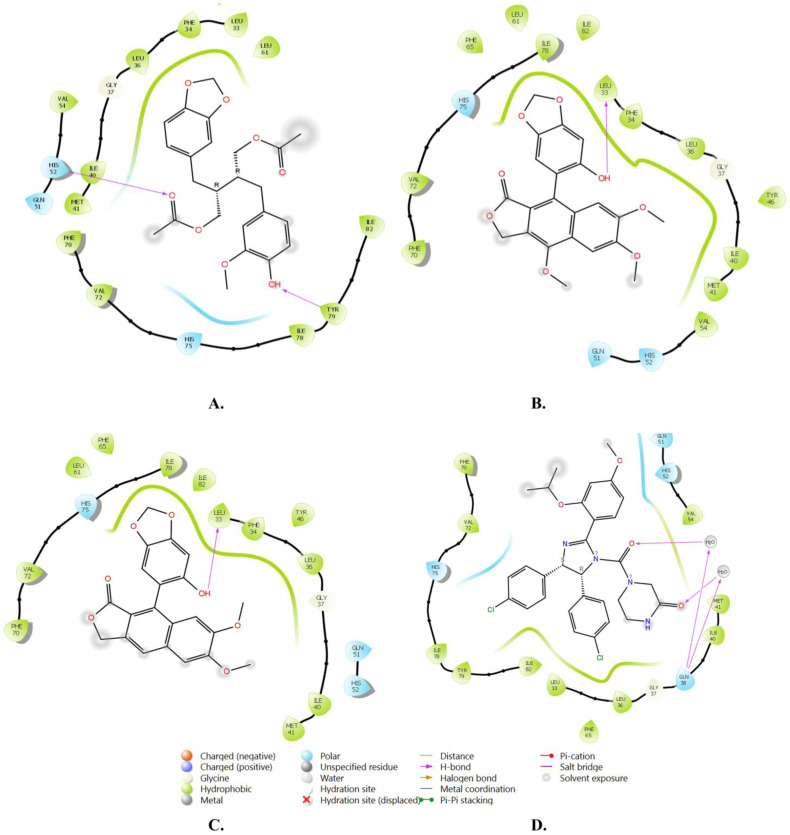
A diagram of the 2D interactions of the selected top three docked ligands and the reference inhibitor with MDM2. The legend below the figure depicts the types of interactions with their color codes. (**A**) Justin A. (**B**) 6′-Hydroxy justicidin A. (**C**) 6′-Hydroxy justicidin B. (**D**) Nutlin-3a.

**Figure 5:**
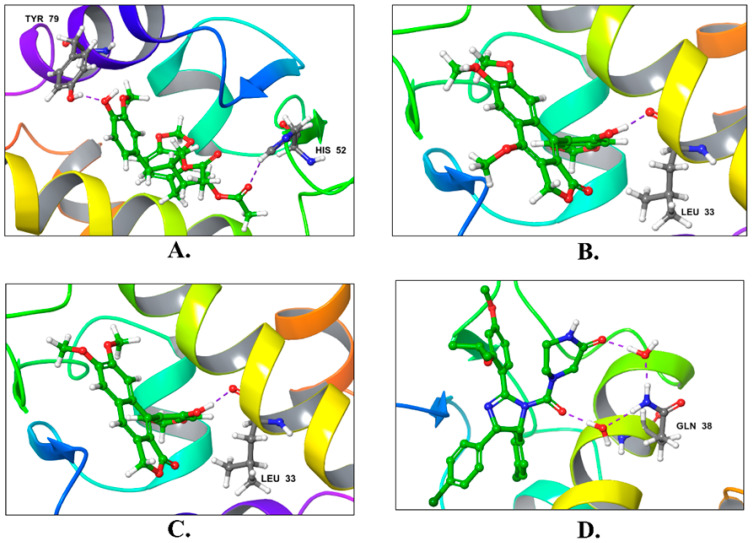
The 3D interactions of the top three docked ligands and the reference with MDM2 (PDB: 5ZXF) using XP Glide. (**A**) Justin A. (**B**) 6′-Hydroxy justicidin A. (**C**) 6′-Hydroxy justicidin B. (**D**) Nutlin-3a. The hydrogen bonds are depicted with violet dashed lines and the three ligands, and the reference is represented with green sticks, while the interacted residues are represented with gray sticks.

Figure 4 and Figure 5 illustrate the two-dimensional and three-dimensional interactions of these compounds in the MDM2 binding domain.

### 2.2. Molecular Dynamics Simulation

To evaluate the behavior of the hit compounds with favorable docking scores and druggable ADMET properties, a molecular dynamics (MD) simulation was performed for 100 ns. The complexes of MDM2 bound to justin A, 6′-hydroxy justicidin A, and 6′-hydroxy justicidin B were simulated using the Desmond package in an explicit TIP3P water model. MD simulations of small-molecule inhibitors with their target proteins provide insights into the system’s flexibility and stability, and the reliability of the binding mode [56].

**Figure 6:**
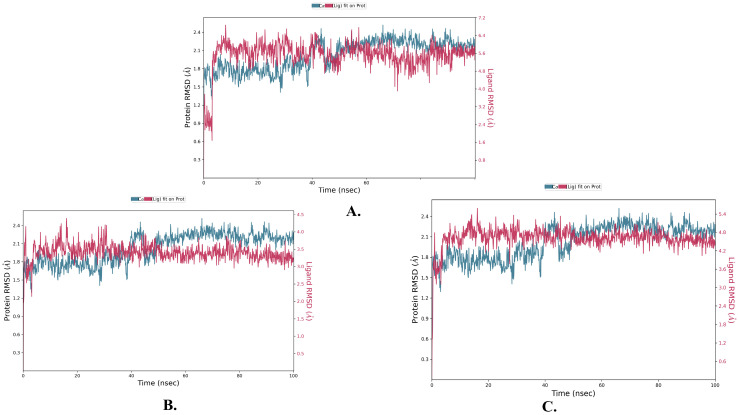
The protein–ligand RMSD plots of the top three ligands in complex with MDM2 (PDB: 5ZXF) during 100 ns simulation using Desmond v6.5 software. (**A**) Justin A. (**B**) 6′-Hydroxy justicidin A. (**C**) 6′-Hydroxy justicidin B.

To assess the stability of the ligand–protein complexes, a root-mean-square deviation (RMSD) analysis was conducted on the simulation trajectories, as shown in Figure 6 and Table 3. 

RMSD measures the deviation of a system from its initial conformation throughout the simulation time and serves as an indicator of system stability. In the case of a globular protein, an acceptable range of RMSD fluctuation typically falls between 1 and 3 Å. If the fluctuations exceed this range, it suggests significant conformational changes within the system during the simulation. A rigorous analysis of the trajectories for the three candidate compounds revealed an acceptable pattern of fluctuation in their RMSD graphs, as depicted in Figure 6. 

The compound justin A exhibited an RMSD pattern with an average of 2.066 ± 0.256 Å. Although it displayed fluctuations within the first 10 ns, it eventually stabilized around the average value. However, regarding 6-hydroxy justicidin A and 6-hydroxy justicidin B, both compounds showed similar fluctuation patterns, converging after approximately 5 ns of simulation, resulting in RMSD averages of 2.042 ± 0.154 and 2.306 ± 0.178 Å, respectively. Notably, the average RMSD for the carbon alpha protein in the three complexes was 2.025 ± 0.252 Å. 

**Figure 7:**
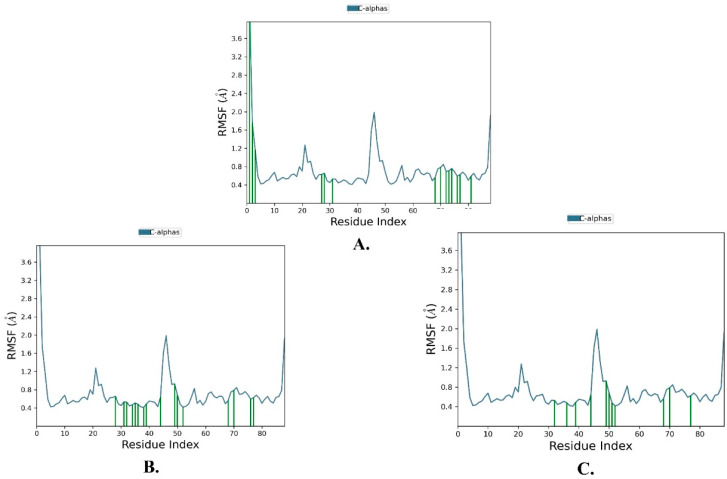
The protein RMSF of MDM2 (PDB: 5ZXF) in complex with the top three ligands during 100 ns simulation using Desmond software. (**A**) Justin A. (**B**) 6′-Hydroxy justicidin A. (**C**) 6′-Hydroxy justicidin B. The green vertical bars indicate the regions of ligand–protein contacts.

An RMSF analysis is a valuable tool for characterizing regional changes in the protein chain, specifically reflecting the stability of individual amino acid residues, particularly those present in the active site. Figure 7 and Table 3 illustrate the RMSF results of the simulated inhibitors, providing insights into the regional changes observed during the simulation. It is important to mention that the average RMSF values (0.721 ± 0.489 Å) remained constant, indicating minimal fluctuations in the ligand–protein contacts and enhanced stability of the protein chain.

The relatively low root-mean-square deviation (RMSD) values of the three candidate compounds, all below 3 Å, and the minimal fluctuations observed in the RMSF plots further support the stability of the complexes.

**Figure 8:**
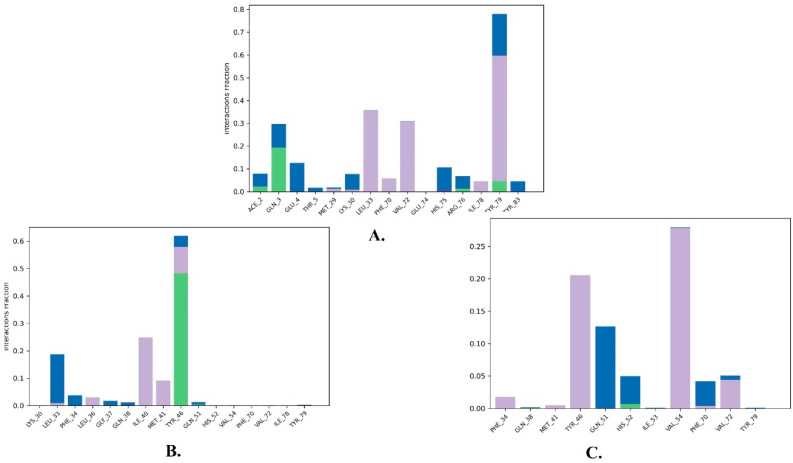
The protein–ligand interactions of the top three ligands with MDM2 (PDB: 5ZXF) during 100 ns MD. (**A**) Justin A. (**B**) 6′-Hydroxy justicidin A. (**C**) 6′-Hydroxy justicidin B.

An extensive analysis of the molecular dynamics (MD) trajectories revealed the interactions between amino acid residues and the simulated ligands throughout the simulation period. The protein residues exhibited various types of interactions with the ligands, including hydrophobic interactions, hydrogen bonds, and water-bridged hydrogen bonds, as depicted in the stacked bar histogram in Figure 8. 

For instance, justin A formed a hydrogen bond with GLN-3 (20%) and displayed hydrophobic contacts with LEU-33 (35%), VAL-72 (30%), and TYR-79 (55%). Additionally, water bridges were observed with GLN-3 (10%), GLU-4 (13%), HIS-75 (10%), and TYR-79 (20%). Regarding the interactions of 6′-hydroxy justicidin A, it mainly engaged in a direct hydrogen bond with TYR-46 throughout approximately 45% of the simulation, as well as a water-bridged hydrogen bond with LEU-33 for nearly 20% of the simulation duration. Additionally, hydrophobic contacts were observed with ILE-40 (25%), MET-41 (10%), and TYR-46 (10%). However, hydroxy justicidin B primarily formed a water bridge with GLN-51 (12%), HIS-52 (5%), and PHE-70 (5%). It also displayed hydrophobic interactions with TYR-46 (20%), VAL-54 (27%), and VAL-72.

A general observation of the protein–ligand interaction results obtained from the MD simulation, in comparison to those obtained from molecular docking, indicates a degree of variability in the types of interactions. This is consistent with the fact that molecular docking considers only a single frame of interaction due to its limited flexibility, while molecular dynamics provides a comprehensive account of all possible interactions across multiple simulation frames.

**Figure 9:**
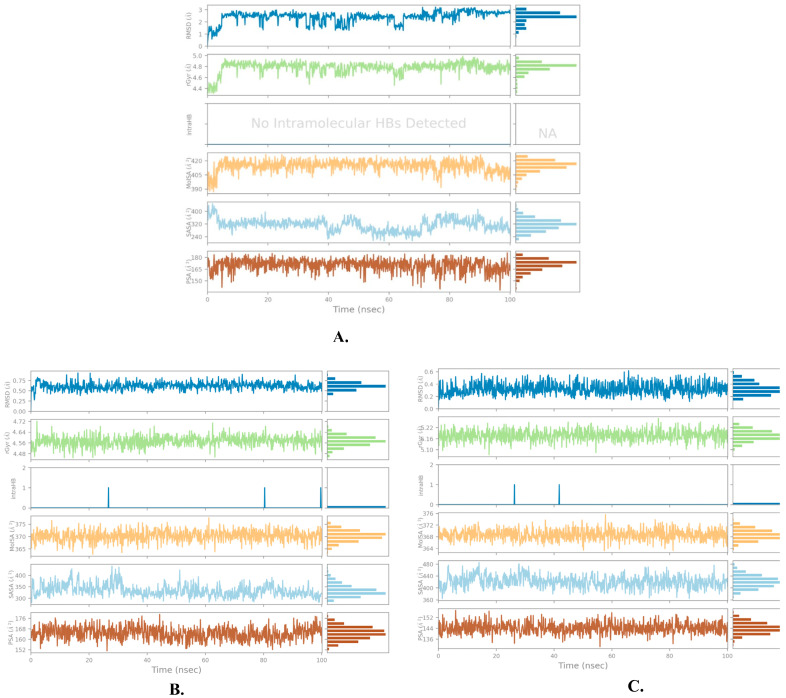
Ligand properties of the top three ligands with MDM2 (PDB: 5ZXF) during 100 ns simulation using Desmond software. (**A**) Justin A. (**B**) 6′-Hydroxy justicidin A. (**C**) 6′-Hydroxy justicidin B.

Further analysis of the ligand properties of the candidate compounds was conducted by means of other measures, such as the radius of gyration (rGyr), molecular surface area (MolSA), solvent accessible surface area (SASA), and polar surface area (PSA). The rGyr descriptor is typically a measurement of the degree of the extendedness of a molecule in relation to its center of mass throughout the simulation; i.e., it accounts for the root-mean-square distance from a molecule’s center of mass. It is calculated in Angstrom units in the MD algorithm used in Desmond. The value explains the pattern of a molecule’s stability in a simulation timeline in the manner of higher values indicating larger flexibility and hence less stability and greater conformational changes of a molecule. As summarized in Figure 9, the values of rGyr fluctuated between 4.4 and 5.0 Å, with an average of 4.77 Å for the justin A compound. Meanwhile, 6′-hydroxy justicidin A had an rGyr value in a range of 4.48–4.72 Å and an average of 4.57 Å. As per 6′-hydroxy justicidin B, it displayed a swing of rGyr between 5.10 and 5.22 Å and an average value of 5.18 Å.

The other studied molecular descriptor is the MolSA, and it is a representation of molecular boundaries that helps in governing the molecular interactions with the surrounding environment and other molecules. In this context, the MolSA given in Figure 9 refers to the Vander Waal surface. It is useful for identifying the area available for steric clashes and other non-bonded interactions. 

Moreover, the solvent-accessible surface area (SASA) is another metric that quantifies the wide-open surface area of a molecule that can be accessed by a solvent system. An SASA analysis gives an insight into a ligand’s binding and protein folding. During a simulation, careful monitoring of SASA changes displays how a molecule’s surface area evolves, which provides potential information about the dynamics of the system and the conformational changes. The average values for justin A, 6′-hydroxy justicidin A, and 6′-hydroxy justicidin B were 313.74, 333.01, and 421 Å^2^, respectively. In the context of a molecular dynamics simulation, the PSA of a molecule is the exposed surface area that possesses charged or polar atoms or functional groups. Importantly, PSA as a descriptor gives information about a molecule’s solubility and permeability, and the potential possible polar interactions. The relevance of PSA’s importance is that, if a molecule is highly polar, it will face difficulties in crossing cellular membranes. Interestingly, the candidate compounds presented acceptable values of PSA averages, as is shown in Figure 9, whereby justin A, 6′-hydroxy justicidin A, and 6′-hydroxy justicidin B gave respective values of 170, 164.59, and 144.19 Å^2^. All the studied ligand properties suggest the stability of the complexes throughout the simulation time.

To further check the quality of the molecular dynamics simulation, post-MD MM/GBSA calculations were carried out for the three candidate compounds. Remarkably, the results are in line with those shown in the molecular docking, whereby the compound justin A gave a lower binding energy of an average −45.32 ± 5.89 kcal/mol. Comparably, 6′-hydroxy justicidin A and 6′-hydroxy justicidin B resulted in binding energies of −35.31 ± 2.68 and −25.43 ± 2.91, respectively. 

## 3. Materials and Methods

In silico studies were performed using Maestro v12.8 from the Schrodinger suite [57]. Molecular dynamics (MD) simulations were conducted using Academic Desmond v6.5 developed by D.E. Shaw Research.

### 3.1. Protein and Ligand Preparation

The crystallographic structure of MDM2 in complex with Nutlin-3a (PDB ID: 5ZXF) was obtained from the Protein Data Bank (PDB) with a resolution of 1.25 Å. The protein structure then underwent various pre-processing and refinement steps using the Protein Preparation Wizard tool in Maestro [58]. These steps included assigning bond orders to untemplated residues, adding explicit hydrogens, creating zero-order bonds to metals and disulfide bonds between close sulfurs, filling in missing side chains and loops, converting selenomethionines to methionines, and generating the most favorable protonation and charge states for heterogroups and residues at a neutral pH. Epik and PROPKA tools were employed for these tasks. Finally, the optimized protein structure was subjected to energy minimization using the OPLS4 force field [59]. 

A group of lignans consisting of 120 structures was collected from a previous publication [30,31] and also subjected to energy minimization using the OPLS4 force field, utilizing the MacroModel tool in Maestro [60] with the PRCG (Polac–Ribiere conjugate gradient) method (2500 iterations). For upcoming studies, the conformation with the lowest energy for each compound was chosen.

### 3.2. Grid Generation and Molecular Docking

After the protein preparation process, the binding sites required for the docking process were determined around the bound ligands using the Receptor Grid Generation panel [61]. This panel uses the coordinates of the bound ligand to establish a precise 3D grid that accurately represents the active site of the protein.

For the molecular docking process, the Glide module within Maestro was employed. Glide offers three distinct docking modes, namely, high-throughput virtual screening (HTVS), standard precision (SP), and extra-precision (XP) modes [39], which differ in speed, accuracy, and scoring function, since HTVS and SP have the same function, while XP uses an extensive sampling and a complex scoring function that penalizes compounds with reduced complementarity with the protein binding cavity [62,63].

### 3.3. ADMET Prediction

The compounds that exhibited docking scores superior to those of Nutlin-3a underwent additional analysis for ADMET (Absorption, Distribution, Metabolism, Excretion, and Toxicity) properties prediction using the QikProp tool within Maestro with the fast mode [64].

### 3.4. MD Simulations and Post-MD MM-GBSA

The MD simulations were conducted using the Desmond platform for the top ligands that were selected based on the obtained ADMET properties results [65]. Initially, the biological system was prepared by immersing the ligand-MDM2 complexes in 4521 TIP3P molecules within an orthorhombic box measuring 10 × 10 × 10 Å [66]. The system was then neutralized by adding 72.389 mM (total charge + 13) of Na^+^ ions and 52.281 mM (total charge-18) of Cl^−^ ions to achieve the physiologic concentration of 150 mM. The SHAKE algorithm was utilized to constrain the motility of all hydrogen bonds [67]. Subsequently, energy minimization was performed utilizing the OPLS4 force field, followed by equilibration using two ensembles: isothermal–isochoric (NVT) and isothermal–isobaric (NPT) ensembles.

The simulation was initiated and conducted for 100 ns and recorded every 100 ps, maintaining a constant temperature of 300 K and an atmospheric pressure of 1 bar. The Particle Mesh Ewald method was used to calculate long-range electrostatic interactions. In Coulomb interactions, the cutoff radius was 9.0 Å. The simple point charge model was utilized for the clear description of water molecules [68,69]. The Nose–Hoover chain thermostat and Martyna–Tobias–Klein barostat were employed for temperature and pressure control, respectively [70,71]. Throughout the simulation, a total of 1002 frames were collected, which were later analyzed using the Simulation Interaction Diagram tool of Desmond. The RMSD for frame X was calculated using the following equation:

$${RMSD}_{x}=\sqrt{{\frac{1}{N}\sum_{i=1}^{N}(r_{i}^{\prime }(t_{x})){-r}_{i}(t_{ref}))}^{2}}$$

where ***N*** is the number of atoms in the atom selection; *t_ref_* is the reference time (typically, the first frame is used as the reference, and it is regarded as time *t* = 0); and *r′* is the position of the selected atoms in a frame *x* after superimposition on the reference frame, where frame *x* is recorded at time *t_x_*. The procedure was repeated for every frame in the simulation trajectory.

The RMSF for residue i was calculated using the following formula:

$${RMSF}_{i}=\sqrt{{\frac{1}{T}\sum_{t=1}^{T}<(r_{i}^{\prime }(t_{x})){-r}_{i}(t_{ref}))}^{2}>}$$

where *T* is the trajectory time over which the RMSF is calculated; *r_i_* is the position of residue I; *r*′ is the position of atoms in residue *i* after superposition on the reference; and the angle brackets indicate that the average of the squared distance is taken over the selection of atoms in the residue.

After MD, the free binding energy of the three ligands was calculated using the MM-GBSA method for the trajectories of the MDM2–ligand complexes via the Prime module of Maestro to further confirm the results. The Prime module of Schrödinger was utilized to calculate the binding free energies of the MD conformations of the complexes using the MM-GBSA continuum solvent model, which incorporates the OPLS4 force field, VSGB solvent model, and rotamer search algorithms [72].

Due to the high computational cost, 6 frames were obtained for all trajectory frames, selecting 1 frame every 200 frames. The following equation was used in the MM-GBSA calculations:

ΔE = E_C_ − E_R_ − E_L_

where ΔE is the free binding energy, Ec is the protein–ligand complex energy, E_R_ is the receptor energy, and E_L_ is the ligand energy. The force field and the solvent model were set to OPLS4 and VSGB, respectively [73].

## 4. Conclusions

MDM2 overexpression is a common characteristic in various types of cancer, allowing cancer cells to evade normal cell division control and promote uncontrolled growth and metastasis. Targeting MDM2, a significant regulator of the tumor suppressor protein p53, presents a promising strategy for developing effective anticancer drugs. In this study, we identified three natural small-molecule inhibitors (justin A, 6-hydroxy justicidin A, and 6′-hydroxy justicidin B) that specifically target the interaction between p53 and MDM2. Justin A, 6-hydroxy justicidin A, and 6′-hydroxy justicidin B are antero-type aryl naphthalide lignans with the lactone carbonyl facing the phenyl group that were reported from *J. procumbens*. Through molecular docking and dynamics simulations, we found that these compounds exhibited strong binding affinities to MDM2, surpassing the binding affinity of the reference inhibitor Nutlin-3a. Moreover, they displayed favorable pharmacokinetic properties and met the criteria for druggability. The molecular dynamics simulations showed stable complex formation between these compounds and MDM2. These findings suggest that these natural compounds hold promise as potential treatments for cancer, after further laboratory testing through in vitro and in vivo investigations. Our study provides a foundation for optimizing and refining these compounds for future therapeutic development. Also, these in silico data provide further evidence for the traditional uses of these natural compounds in cancer treatment.

## Figures and Tables

**Table 1:** Docking scores, hydrogen bonding, and hydrophobic interactions of the top-docked complexes.

Compound	Title	Docking Score Kcal/mol	H-Bonding	Hydrophobic Interactions
1	Justin A	−7.526	HIS-52 (2.73 Å) TYR-79 (1.97 Å)	LEU-33, ILE-40, MET-41, PHE-65, PHE-70, VAL-72, ILE-78, TYR-79
2	6′-Hydroxy justicidin A	−7.438	LEU-33 (1.87 Å)	LEU-33, LEU-36, ILE-40, MET-41, PHE-65, PHE-70, VAL-72, ILE-78
3	6′-Hydroxy justicidin B	−7.240	LEU-33 (1.94 Å)	LEU-33, LEU-36, ILE-40, MET-41, PHE-65, PHE-70, VAL-72, ILE-78
4	Lariciresinol	−7.067	Bridged H-bond with PHE-34 GLN-51 (1.88 Å) HIS-52 (2.44 Å) TYR-79 (2.36 Å)	LEU-33, VAL-72, TYR-79
5	Procumbiene	−7.027	Bridged H-bond with GLN-38	LEU-33, ILE-40, PHE-65, PHE-70, VAL-72, ILE-78, TYR-79
6	Diphyllin	−6.985	-	LEU-33, LEU-36, ILE-40, MET-41, PHE-65, PHE-70, VAL-72, ILE-78
7	6′-Hydroxy justicidin C	−6.966	LEU-33 (1.90 Å)	LEU-33, LEU-36, ILE-40, MET-41, PHE-65, PHE-70, VAL-72, ILE-78
8	(+)-Sinkianlignan E	−6.877	TYR-79 (2 Å) GLN-51 (1.69 Å)	LEU-33, ILE-40, PHE-65, PHE-70, VAL-72, ILE-78, TYR-79
9	Pinoresinol	−6.831	TYR-79 (2.18 Å) GLN-51 (1.87 Å) HIS-52 (2.5, 2.8 Å)	LEU-33, ILE-40, VAL-72, ILE-78, TYR-79
**Reference**	Nutlin-3a	−6.830	2 bridged H-bonds with GLN-38	LEU-33, LEU-36, ILE-40, MET-41, PHE-65, VAL-72, ILE-78, TYR-79

**Table 2:** The pharmacokinetic profile of the top-docked candidates.

Compound	Donor HB ^a^	Accpt HB ^b^	QPlog Po/w ^c^	QPlog S ^d^	QPlog HERG ^e^	QPP Caco ^f^	QPlog BB ^g^	Mwt ^h^	% HOR ^i^	ROF ^j^
Justin A	1	7	4.074	−4.924	−5.086	380.953	−1.546	444.480	96.9	0
6′−Hydroxy justicidin A	1	7	2.617	−3.414	−4.082	1497.331	−0.481	410.379	100	0
6′−Hydroxy justicidin B	1	7	2.561	−3.384	−4.160	1471.994	−0.417	380.353	100	0
Lariciresinol	3	6	2.651	−3.815	−4.919	528.187	−1.169	360.406	91	0
Procumbiene	1	8	1.693	−2.004	−3.387	1121.839	−0.494	368.342	91	0
Diphyllin	1	7	2.528	−3.383	−4.122	1332.149	−0.455	380.353	100	0
6′−Hydroxy justicidin C	1	7	2.532	−3.339	−4.005	1275.549	−0.537	410.379	100	0
(+)−Sinkianlignan E	2	7	3.791	−4.375	−5.514	984.675	−1.139	358.433	100	0
Pinoresinol	2	6	2.849	−4.393	−4.917	969.614	−0.669	358.390	100	0
Nutlin−3a (reference)	1	7	5.098	−5.968	−2.904	296.106	−0.288	581.497	75	2
Standard values	≤5	≤10	−2.0–6.5	−6.5–0.5	Below −5	>25 poor <500 great	−3–1.2	>500	>25% poor <80% great	0–4

Note: ^a^ hydrogen bond donor. ^b^ Hydrogen bond acceptors. ^c^ Predicted octanol/water partition coefficient. ^d^ Predicted aqueous solubility. ^e^ Predicted IC50 values for the blockage of HERG K^+^. ^f^ Predicted cell permeability by a model of Caco−2 cells. ^g^ Predicted brain/blood partition coefficient. ^h^ Molecular weight. ^i^ Percentage of human oral absorption. ^j^ Number of violations of Lipinski’s rule of five.

**Table 3:** The average values of RMSD and RMSF for the top 3 candidate compounds.

Name	RMSD		RMSF of Cα
	Cα	Ligand with Protein	
Justin A	2.025 ± 0.252	2.066 ± 0.256	0.721 ± 0.489
6′-Hydroxy justicidin A	2.025 ± 0.252	2.042 ± 0.154	0.721 ± 0.489
6′-Hydroxy justicidin B	2.025 ± 0.252	2.306 ± 0.178	0.721 ± 0.489

## Data Availability

The original contributions presented in the study are included in the article. Further inquiries can be directed to the corresponding author.

## Supplementary Materials

**Table S1.** List of *Justica Procumbens* and *Ferula sinkiangensis* lignans. The following supporting information can be downloaded at: https://www.mdpi.com/article/10.3390/molecules28186665/s1.
