# Supplementary material for: Molecular Docking and Molecular Dynamics Studies Reveal the Anticancer Potential of Medicinal-Plant-Derived Lignans as MDM2-P53 Interaction Inhibitors
Source: Molecules. 2023 Sep 16;28(18):6665. doi: 10.3390/molecules28186665 (PMC10536213; doi:10.3390/molecules28186665)
Supplement: Supplementary file 1 [file molecules-28-06665-s001.zip › molecules-2605754-supplementary.pdf]

Supplementary Materials

Molecular Docking and Molecular Dynamics Studies Reveal the Anticancer Potential of Medicinal-Plant-Derived Lignans as MDM2-P53 Interaction Inhibitors

Tagyedeen H. Shoaib [1,†], Nihal Abdelmoniem [1], Rua M. Mukhtar [1], Amal Th. Alqhtani [2], Abdullah L. Alalawi [3], Razan Alawaji [4], Mashael S. Althubyani [2], Shaimaa G. A. Mohamed [5], Gamal A. Mohamed [6], Sabrin R. M. Ibrahim [7,8,*], Hazem G. A. Hussein [9] and Abdulrahim A. Alzain [1,†,*]

[1] Department of Pharmaceutical Chemistry, Faculty of Pharmacy, University of Gezira, Wad Madani 21111, Sudan; shoaibth37@hotmail.com (T.H.S.); nihal.khunaijir@gmail.com (N.A.); ruamubarak1@gmail.com (R.M.M.)

[2] Pharmaceutical Care Services, Madinah Cardiac Center, MOH, Al Madinah Al Munawwarah 11176, Saudi Arabia; amtalqhtani@moh.gov.sa (A.T.A.); maalthubyani@moh.gov.sa (M.S.A.)

[3] Pharmaceutical Care Services, King Salman Medical City, MOH, Al Madinah Al Munawwarah 11176, Saudi Arabia; abdullahalalawi96@gmail.com

[4] Department of Pharmacology and Toxicology, College of Pharmacy, Qassim University, Qassim 51452, Saudi Arabia; razanalawaji@gmail.com

[5] Faculty of Dentistry, British University, El Sherouk City, Suez Desert Road, Cairo 11837, Egypt; shaimaag1973@gmail.com

[6] Department of Natural Products and Alternative Medicine, Faculty of Pharmacy, King Abdulaziz University, Jeddah 21589, Saudi Arabia; gahussein@kau.edu.sa

[7] Preparatory Year Program, Department of Chemistry, Batterjee Medical College, Jeddah 21442, Saudi Arabia

[8] Department of Pharmacognosy, Faculty of Pharmacy, Assiut University, Assiut 71526, Egypt

[9] Preparatory Year Program, Batterjee Medical College, Jeddah 21442, Saudi Arabia; hazemgamal2005@gmail.com

* Correspondence: sabrin.ibrahim@bmc.edu.sa or sabreen.ibrahim@pharm.aun.edu.eg (S.R.M.I.); abdulrahim.altoam@uofg.edu.sd or abdulrahim.altoam@gmail.com (A.A.A.)

† These authors have contributed equally.

Table S1. List of *Justica Procumbens* and *Ferula sinkiangensis* lignans

**Compound Name**	**Mol. Wt.**	**Mol. Formula**
Justicidin A	394	$C_{22}H_{18}O_7$
6`-Hydroxy justicidin A	410	$C_{22}H_{18}O_8$
Justicidin B	364	$C_{21}H_{16}O_6$
6`-Hydroxy justicidin B	380	$C_{21}H_{16}O_7$
Neojusticin B = Justicidin C	394	$C_{22}H_{18}O_7$
Neojusticin A = Justicidin D	378	$C_{21}H_{14}O_7$
6`-Hydroxy justicidin C	410	$C_{22}H_{18}O_8$
Neojusticin C	380	$C_{21}H_{16}O_7$
Justicidin E	348	$C_{20}H_{12}O_6$
Phyllamyricin C	394	$C_{22}H_{18}O_7$
Taiwanin C = Lignan J_1	378	$C_{21}H_{14}O_7$
Taiwanin E	364	$C_{20}H_{12}O_7$
Taiwanin E methyl ether	378	$C_{21}H_{14}O_7$
Chinensinaphthol	380	$C_{21}H_{16}O_7$
Chinensinaphthol methyl ether	394	$C_{22}H_{18}O_7$
4`-Demethylchinensinaphthol methyl ether	380	$C_{21}H_{16}O_7$
Procumphthalide A	394	$C_{22}H_{18}O_7$
Cilinaphthalide A	396	$C_{22}H_{20}O_7$
Cilinaphthalide B	410	$C_{23}H_{22}O_7$
Pronaphthalide A	396	$C_{22}H_{20}O_7$
5`-Methoxy retrochinensin	394	$C_{22}H_{18}O_7$
Diphyllin	380	$C_{21}H_{16}O_7$
Isodiphyllin	380	$C_{21}H_{16}O_7$
Tuberculatin (Diphyllin-1-O-β-D-apiofuranoside)	512	$C_{26}H_{24}O_{11}$
Diphyllin apioside-5-acetate	554	$C_{28}H_{26}O_{12}$
Justicidinoside A (Justicidin C 6`-O-glucopyranoside)	572	$C_{28}H_{28}O_{13}$
Justicidinoside B (Justicidin A 6`-O- glucopyranoside)	572	$C_{28}H_{28}O_{13}$
Justicidinoside C (Justicidin B 6`-O- glucopyranoside)	542	$C_{27}H_{26}O_{12}$
Procumbenoside A (4-O-α-L-Arabinopyranosyl-(1```→ 2``)- β-D-apiofuranosyldiphyllin	644	$C_{31}H_{32}O_{15}$
Procumbenoside B = Procumphthalide B (4-O-β-D-Gluco pyranosyl-(1```→2``)-β-D-apiofuranosyldiphyllin)	674	$C_{32}H_{34}O_{16}$
Procumbenoside C	526	$C_{26}H_{22}O_{12}$
Procumbenoside D	542	$C_{27}H_{26}O_{12}$

Procumbenoside H (4-O-β-D-Xylopyranosyl-(1``` → 5``)-β-D-apiofuranosyl diphyllin)	644	$C_{31}H_{32}O_{15}$
Procumbenoside E (4-O-β-D-Bis-xylopyranosyl-(1``` → 5``, 1```→2``)-β-D-apiofuranosyl diphyllin)	776	$C_{36}H_{40}O_{19}$
Procumbenoside I	528	$C_{26}H_{24}O_{12}$
Procumbenoside J	552	$C_{29}H_{28}O_{11}$
Procumbenoside K	532	$C_{26}H_{28}O_{12}$
Procumbenoside L	502	$C_{25}H_{26}O_{11}$
Procumbenoside M	738	$C_{37}H_{38}O_{16}$
Procumbenoside N (7-O-[β-D-Xylopyranosyl-(1```→2``)-β-D-glucopyranosyl]-diphyllin)	674	$C_{32}H_{34}O_{16}$
Procumbenoside O (7-*O*-[β-D-Glucopyranosyl-(1````→5``)-β-D-xylopyranosyl-(1```→2``)-β-D-apiofuranosyl]-diphyllin)	806	$C_{37}H_{42}O_{20}$
Cleistanthin B	542	$C_{27}H_{26}O_{12}$
Ciliatoside A (4-O-[*α*-L-Arabinopyranosyl-(1```→2``)-β-D-xylopyranosyl-(1````→5``)-β-D-apiofuranosyl]diphyllin)	776	$C_{36}H_{40}O_{19}$
Ciliatoside B (4-*O*-{[*β*-D-Apiofuranosyl-(1`````→3```)-*α*-L-arabinopyranosyl-(1```→2``)][*β*-D-xylopyranosyl-(1````→5``)]-*β*-D-apiofuranosyl}diphyllin)	908	$C_{41}H_{48}O_{23}$
Azizin (4-O-[β-D-Xylopyranosyl-(1```→2``)-β-D-xylopyranosyl-(1````→5``)-β-D-apiofuranosyl]diphyllin)	776	$C_{36}H_{40}O_{19}$
Justatropmer A (4-O-β-D-Apiofuranosyl-6′-hydroxydiphyllin)	528	$C_{26}H_{24}O_{12}$
Justatropmer B (4-O-β-D-apiofuranosyl-6′-hydroxydiphyllin)	528	$C_{26}H_{24}O_{12}$
Justatropmer C (4-O-[β-D-Xylopyranosyl-(1→2)-β-D-apiofuranosyl]-6′-hydroxydiphyllin)	660	$C_{31}H_{32}O_{16}$
Justatropmer D (4-O-[β-D-Xylopyranosyl-(1→2)-β-D-apiofuranosyl]-6′-hydroxydiphyllin)	660	$C_{31}H_{32}O_{16}$
Justatropmer E (4-O-[Bis-β-D-xylopyranosyl-(1→2, 1→5)-β-D- apiofuranosyl]-6′-hydroxydiphyllin)	792	$C_{36}H_{40}O_{20}$
Justatropmer F (4-O-[Bis-β-D-xylopyranosyl-(1→2, 1→5)-β-D- apiofuranosyl]-6′-hydroxydiphyllin)	792	$C_{36}H_{40}O_{20}$
Justatropmer G (4-O-{[β-D-Apiofuranosyl-(1→3)-β-D-xylopyranosyl-(1→2)][β-D-xylopyranosyl-(1 → 5)]-β-D-apiofuranosyl}-6′-hydroxydiphyllin)	924	$C_{41}H_{48}O_{24}$
Justatropmer H (4-O-{[β-D-Apiofuranosyl-(1→3)-β-D-xylopyranosyl-(1→2)][β-D-xylopyranosyl-(1 → 5)]-β-D-apiofuranosyl}-6′-hydroxydiphyllin)	924	$C_{41}H_{48}O_{24}$
Justatropmer I (6′-O-β-D-Glucopyranosyldiphyllin)	558	$C_{27}H_{26}O_{13}$
Justatropmer J (4-O-β-D-Glucopyranosyl-6′-hydroxyl-diphyllin)	558	$C_{27}H_{26}O_{13}$
Justatropmer K (4-O-β-D-Glucopyranosyl-6′-hydroxyl-diphyllin)	558	$C_{27}H_{26}O_{13}$

Justatropmer L (6`-O-[β-D-6-O-Acetylglucopyranosyl]-justicidin B)	584	$C_{29}H_{28}O_{13}$
Justatropmer M (6`-O-β-D-Glucopyranosyl-retro-justicidin A)	572	$C_{28}H_{28}O_{13}$
Justin A	444	$C_{24}H_{28}O_{8}$
Justin B	474	$C_{25}H_{30}O_{9}$
Justin C	490	$C_{26}H_{34}O_{9}$
(-)-Dihydroclusin	404	$C_{22}H_{28}O_{7}$
(-)-Dihydroclusin diacetate	488	$C_{26}H_{32}O_{9}$
Secoisolariciresinol	362	$C_{20}H_{26}O_{6}$
Secoisolariciresinol dimethyl ether	390	$C_{22}H_{30}O_{6}$
Secoisolariciresinol dimethyl ether monoacetate	432	$C_{24}H_{32}O_{7}$
Secoisolariciresinol dimethyl ether diacetate	474	$C_{26}H_{34}O_{8}$
5-Methoxy-4,4`-di-*O*-methylsecolariciresinol	420	$C_{23}H_{32}O_{7}$
5-Methoxy-4,4`-di-O-methylsecolariciresinol monoacetate	462	$C_{25}H_{34}O_{8}$
5-Methoxy-4,4`-di-*O*-methylsecolariciresinol diacetate	504	$C_{27}H_{36}O_{9}$
2,3-Demethoxysecisolintetralin acetate	458	$C_{25}H_{30}O_{8}$
Hemiariensin	400	$C_{22}H_{24}O_{7}$
Ariensin	442	$C_{24}H_{26}O_{8}$
Rostellulin A	472	$C_{25}H_{28}O_{9}$
Hinokinin	354	$C_{20}H_{18}O_{6}$
Procumbiene	368	$C_{20}H_{16}O_{7}$
Juspurpudin	368	$C_{20}H_{16}O_{7}$
Pinoresinol	372	$C_{21}H_{24}O_{6}$
(-)-Syringaresinol	418	$C_{22}H_{26}O_{8}$
Lariciresinol	360	$C_{20}H_{24}O_{6}$
5`-Methoxy-4`-O-methyllariciresinol	418	$C_{23}H_{30}O_{7}$
(7`*S*,8`*R*)-5,3`-Dimethoxy-4,4`,9`-trihydroxy-2-8`,9-*O*-7`-neolignan	360	$C_{20}H_{24}O_{6}$
Arctigenin	372	$C_{21}H_{24}O_{6}$
Matairesinol	358	$C_{20}H_{22}O_{6}$
Secroisolariciresinol	362	$C_{20}H_{26}O_{6}$
(7,8-cis-8,8`-trans)-2-4-Dihydroxyl-3,5-dimethoxy-lariciresinol	360	$C_{20}H_{24}O_{6}$
(+)-Ferulasinkin A (7R, 8R, 7`R, 8`R)	362	$C_{19}H_{22}O_{7}$
(-)-Ferulasinkin A (7S, 8S, 7`S, 8`S)	362	$C_{19}H_{22}O_{7}$
(+)-Ferulasinkin B (7S, 8S, 7`R, 8`R)	362	$C_{19}H_{22}O_{7}$
(-)-Ferulasinkin B (7R, 8R, 7`S, 8`S)	362	$C_{19}H_{22}O_{7}$
(+)-Ferulasinkin C (7S, 8S, 7`R, 8`S)	362	$C_{19}H_{22}O_{7}$
(-)-Ferulasinkin C (7R, 8R, 7`S, 8`R)	362	$C_{19}H_{22}O_{7}$

(+)-Ferulasinkin D (7R, 8S, 7`S, 8`S)	362	$C_{19}H_{22}O_7$
(-)-Ferulasinkin D (7S, 8R, 7`R, 8`R)	362	$C_{19}H_{22}O_7$
(+)-Sinkianlignan A (7``S, 8``R)	518	$C_{30}H_{30}O_8$
(-)-Sinkianlignan A (7``R, 8``S)	518	$C_{30}H_{30}O_8$
(+)-Sinkianlignan B (7S, 8S, 7``S, 8``R)	552	$C_{30}H_{32}O_{10}$
(-)-Sinkianlignan B (7R, 8R, 7``R, 8``S)	552	$C_{30}H_{32}O_{10}$
(+)-Sinkianlignan C (7R, 8R, 9S, 7``R)	524	$C_{30}H_{36}O_8$
(-)-Sinkianlignan C (7S, 8S, 9R, 7``S)	524	$C_{30}H_{36}O_8$
(+)-Sinkianlignan D (7S, 8R, 9``S)	430	$C_{23}H_{26}O_8$
(-)-Sinkianlignan D (7R, 8S, 9``R)	430	$C_{23}H_{26}O_8$
(+)-Sinkianlignan E (7S, 85)	358	$C_{21}H_{26}O_5$
(-)-Sinkianlignan E (7R, 8R)	358	$C_{21}H_{26}O_5$
(+)-Sinkianlignan F (7R, 85)	358	$C_{21}H_{26}O_5$
(-)-Sinkianlignan F (7S, 8R)	358	$C_{21}H_{26}O_5$
(+)-Sinkianlignan G (7R, 8S, 7`R, 8`S)	376	$C_{20}H_{24}O_7$
(-)-Sinkianlignan G (7S, 8R, 7`S, 8`R)	376	$C_{20}H_{24}O_7$
(+)-Sinkianlignan H (7S, 8R, 7`R, 8`R)	376	$C_{20}H_{24}O_7$
(-)-Sinkianlignan H (7R, 8S, 7`S, 8`S)	376	$C_{20}H_{24}O_7$
(+)-Sinkianlignan I (7R, 8R, 7`S, 8`S)	376	$C_{20}H_{24}O_7$
(-)-Sinkianlignan I (7S, 8S, 7`R, 8`R)	376	$C_{20}H_{24}O_7$
(+)-Sinkianlignan J (7R, 8R, 7`R, 8`R)	376	$C_{20}H_{24}O_7$
(-)-Sinkianlignan J (7S, 8S, 7`S, 8`S)	376	$C_{20}H_{24}O_7$
(+)-Sinkianlignan K (7R, 8S, 7`S, 8`R)	376	$C_{20}H_{24}O_7$
(-)-Sinkianlignan K (7S, 8S, 7`R, 8`S)	376	$C_{20}H_{24}O_7$
Neoarctin A	742	$C_{42}H_{46}O_{12}$
Arctiin	534	$C_{27}H_{34}O_{11}$
